# Adherence to mHealth quit smoking application ‘stopcoach’ on top of evidence based smoking cessation counselling: association with participant characteristics and long-term abstinence

**DOI:** 10.1186/s13011-025-00672-8

**Published:** 2025-10-14

**Authors:** Anne Zijp, Daan L. de Frel, Bas van den Putte, Eline Heemskerk, Niels H. Chavannes, Sander Hermsen, Sigrid Troelstra, Eline Meijer

**Affiliations:** 1 Public Health and Primary Care, Leiden University Medical Center, Leiden, Netherlands; 2 Department of Cardiology, Leiden University Medical Center, Leiden, Netherlands; 3 Amsterdam School of Communication Research, University of Amsterdam, Amsterdam, The Netherlands; 4 Pharos Dutch Center of Expertise On Health Disparities, Utrecht, The Netherlands; 5 National eHealth Living Lab, Leiden University Medical Center, Leiden, Netherlands; 6 OnePlanet Research Center, Imec NL, Wageningen, The Netherlands; 7 The Netherlands Expertise Centre for Tobacco Control, Trimbos Institute, Utrecht, The Netherlands

**Keywords:** Smoking cessation, Socioeconomic position, MHealth, StopCoach, Adherence, User characteristics

## Abstract

**Background:**

Smoking is more common in lower socioeconomic position (SEP) groups, but smoking cessation interventions are less effective for these groups. StopCoach, an mHealth intervention, supports people with lower SEP in quitting smoking. A non-randomized controlled trial found that adding StopCoach to accredited smoking cessation counselling (SCC) led to higher abstinence rates immediately after and one year after SCC, compared to SCC alone. Users also rated its usability, acceptability, and practicality positively. Recent studies show a dose-response relationship, where more mHealth use improves smoking cessation outcomes. Since adherence is a challenge and often underreported, understanding adherence factors is crucial in evaluating eHealth intervention effectiveness.

**Aim:**

This study aims to assess the association between participant characteristics and StopCoach app adherence, as well as the association between app adherence and (short- and long-term) abstinence.

**Methods:**

Main outcomes are app adherence and abstinence. All participants (*N* = 132) were enrolled in group-based SCC. The association between user characteristics and adherence was estimated with logistic regression models. Chi-square tests were performed to test the association between app adherence and self-reported smoking abstinence. Short-term and long-term abstinence were defined as 4 weeks and 1 year after the quit date, respectively. App adherence was defined as the number of steps completed by a participant in the app.

**Results:**

Older users and those who had attended all SCC meetings were significantly more likely to be adherent to the app. More app-adherent participants were significantly more likely to be abstinent 1 year after the quit date. This association was non-significant at 4 weeks.

**Conclusion:**

The results indicate that mHealth interventions such as StopCoach may increase the effectiveness of traditional SCC programs. Users could benefit from further integration of StopCoach in SCC programs, as this could motivate them to be more adherent to StopCoach, resulting in them quitting successfully.

## Introduction

Smoking is the leading cause of preventable death, causing more than 7 million deaths annually worldwide [1]. Although smoking prevalence has declined, 18.9% of Dutch adults still smoked in 2022 [2]. Smoking is most prevalent in lower socioeconomic position (SEP) groups, defined by factors like low education and income, with the exact definition varying by context, and people with a lower SEP who smoke are less likely to quit successfully [3–6]. Moreover, current smoking policies, such as tax increases and smoking bans, are less effective for people with a lower SEP [7–10]. As a result, smoking increases health-related inequities, in particular that people with lower-SEP die 7 years earlier and live almost 20 years shorter in good health than people with higher-SEP [11]. In 2021, 30.9% of Dutch smokers made a serious quit attempt, but the success rate for unassisted attempts after one year was only about 5%, while assisted attempts had a success rate of 20–25% [4]. For people with lower SEP the percentage of a seriously quit attempt is even lower (26.3%) [4]. Evidence-based interventions such as pharmacotherapy and individual or group behavioural smoking cessation counselling (SCC) can increase individual success rates [12].

In recent years, the number of eHealth applications has grown rapidly, offering an accessible, cheap, and constant guide in many health behaviour change processes. Reviews and clinical guidelines for smoking cessation highlight eHealth as a promising approach, especially when eHealth interventions are personalized, interactive, and provide text messages [13–16]. mHealth interventions, a subset of eHealth interventions, refers specifically to health interventions delivered through mobile devices, such as smartphones and tablets, including smoking cessation apps. While smoking cessation apps are abundant, most are not evidence-based, and some even contradict clinical guidelines [17]. In addition, many eHealth evaluation studies lack long-term data (i.e., after one year), and the interventions offered often do not meet the needs of lower SEP populations [18]. However, evidence supports the positive effect of smoking cessation mHealth interventions [15].

Recent studies found a dose-response relationship wherein more mHealth app use positively affected smoking cessation outcomes such as higher cessation rates and higher self-efficacy to quit smoking over time [19–21]. However, adherence rates to smoking cessation apps are often low and variable, and data on adherence is barely reported in studies on the effect of mHealth smoking cessation interventions [22–24]. Also, little research has been done on the association between user characteristics of participants with the amount of use of an mHealth smoking cessation application. A study into a smoking cessation app found that older app users and those who felt quitting was important used the app more [21]. On the other hand, app users who were male users, those who worked, smoked heavy (according to the Heaviness of Smoking Index), or had higher self-efficacy used the app less [21]. Another study showed that lower educational levels, higher nicotine dependence, and more previous quit attempts were associated with greater intention to use a smoking cessation app, while other demographic factors were not [25]. Therefore, considering adherence in eHealth intervention studies is crucial to understanding its effectiveness. This paper examines adherence to and effectiveness of the StopCoach app, inspired by the previously developed StopAdvisor website.

StopAdvisor is an interactive, tailored internet-based intervention for smoking cessation that was targeted at people with lower SEP who smoke [26]. It was developed and evaluated in the United Kingdom with end-users (adults who want to quit smoking), and a randomized controlled trial showed that the interactive ‘StopAdvisor’ website was more effective compared to a non-interactive static website for people with lower SEP who smoke, but not for people with higher SEP who smoke [26]. The StopCoach app provides people who smoke with step-by-step evidence-based information about smoking cessation. StopCoach was developed and evaluated positively by healthcare professionals and people with a lower SEP who smoke in a pilot study in five Dutch municipalities in 2019–2020 [27]. Results of this pilot study showed that app use of StopCoach was higher among people who used the app alongside SCC provided by a professional, and app users were more likely to attempt to quit. Nevertheless, overall low app use was found. The implementation of the app appeared hindered by SCC counsellors’ insufficient preparation and familiarity with the app, and possibly by the perception that integration of the app with SCC was not their task [28].

Subsequently, this was followed by a 2020–2022 study of the effectiveness and acceptability of using StopCoach as an addition to group-based SCC. The first paper on this study reported that when comparing the StopCoach intervention group to a control group receiving SCC (without StopCoach) during the COVID-19 pandemic, abstinence rates were higher in the intervention group after four weeks (73.6% vs. 57.1%) and after one year (34.7% vs. 27.9%) [29]. The intervention group had lower abstinence rates at four weeks compared to the pre-COVID control group (73.6% vs. 78.2%). This effect was attributed to an overall decrease in the effectiveness of the group-based SCC program during the COVID-19 pandemic compared to the pre-COVID period. In line with this, app users rated usability, acceptability, and usefulness highly positive. Qualitative interviews showed that SCC trainers welcomed adding StopCoach to their program. This second paper on the same study will consider the adherence of participants to StopCoach and the effect on smoking cessation of using StopCoach in addition to regular SCC. Therefore, this study will address the following research questions:

1. What is the association between participant characteristics (gender, age, educational level, pack years, medical or psychiatric diagnosis) and StopCoach app adherence?
2. What is the association between StopCoach app adherence and short-term (i.e., four weeks after the quit date) and long-term (i.e., one year after the quit date) abstinence?

## Methods

### Design

This study is part of a non-randomized trial conducted in the Netherlands in collaboration with SineFuma, a provider of evidence-based SCC which is reimbursed through healthcare insurance. The inclusion period for the intervention group used in this study was from December 2020 until June 2022. Initially, intervention group participants were only recruited from the Dutch provinces Zuid-Holland and Noord-Brabant. Due to COVID-19, data collection did not progress as planned, leading to the decision in December 2021 to recruit participants from all Dutch provinces. The SCC lasts 7 weeks, while StopCoach continues for up to 12 weeks. More methodological details can be found in the first publication on this study [29].

### Participants

Eligibility criteria for intervention group participants were: (1) being aged 18 years or older, (2) being a current smoker, (3) being enrolled in SCC at SineFuma, (4) being capable of using, and in possession of, a smartphone or tablet, (5) being able to understand and read Dutch at B1 level (level of StopCoach), (6) willing to use StopCoach. Although StopCoach is designed to be suitable for lower SEP users, it can be used by anyone who want to quit smoking regardless of SEP. Having a lower SEP is therefore not an inclusion criterion. Potential participants were lost to follow-up if they dropped out from SineFuma’s SCC program before the third meeting (planned quit date), as they did not have a defined quit date and had insufficient exposure to the SCC component to meaningfully interpret the combined use of SCC and the app. Of the 226 intervention group participants who provided a phone number in the correct format, only 132 numbers could be linked to StopCoach user data that was provided by the app developer. No technical or other causes could be found for study participants missing in the log data set (see Analyses and Results for the attrition analysis).

### Procedure

As part of standard procedures, SineFuma sends their participants an e-mail before the SCC program starts. For the intervention group, participants were invited by SineFuma through a digital invitation, which they received, as an addition to the standard SCC e-mail, before the start of their SCC group. In addition, coaches were instructed to mention StopCoach in a group’s first SCC meeting. Sinefuma coaches were given a clickthrough version of StopCoach to familiarize themselves with the intervention. Interested SCC participants could request an information letter and informed consent form through the digital invitation. After signing informed consent, participants were included in the study and actively involved for 12 weeks. As part of the standard SineFuma SCC, participants completed a questionnaire with demographic data and smoking characteristics before the start of the SCC. They followed SCC from SineFuma as usual and used StopCoach. For 12 weeks, user data of StopCoach was collected by the application. The abstinence rates after four weeks and one year were also tracked as standard by SineFuma.

### Intervention

The intervention consisted of StopCoach, a smoking cessation application aimed at people with a low SEP who want to quit smoking and was developed by a consortium of Leiden University Medical Center/National eHealth Living Lab, Trimbos-institute (independent, scientific institute on mental health, alcohol, tobacco, and drugs) and Pharos (Dutch Centre of Expertise on Health Disparities). StopCoach was inspired by StopAdvisor, which was originally developed and evaluated in a research collaboration led by a team at University College London. People with lower SEP who smoke, and healthcare professionals were also involved in translating and developing StopAdvisor into StopCoach.

The application provides people who smoke with step-by-step evidence-based information and tips before and during the quit attempt. StopCoach also provides motivational messages from a virtual coach, which users can choose to be male or female. The app also displays money saved and the number of cigarettes not smoked by users. Users can earn stars for completing sub-steps and access audio and video content featuring ex-smokers’ experience with quitting. Additionally, the app includes a quick link to the national smoking cessation telephone quit line, which is hosted by the Trimbos Institute.

StopCoach consists of several consecutive phases, including (1) a preparation phase until the actual quit date set by the user, (2) more intensive support phase after quit date with multiple steps per week, (3) and a less intensive support phase with weekly steps. Users could always consult previous steps, and the app contained a ‘tips & exercises’ section (e.g., providing tips for dealing with stress) that could be accessed at any time. Based on the findings of the previous pilot study, the duration of StopCoach was extended from eight to twelve weeks. Before the start of the study a SCC trainer reviewed StopCoach for integration with SCC, and no changes were deemed necessary. More information on the StopCoach intervention can be found in Meijer et al. (2021) [27].

### Measures

#### Participant demographics

SineFuma provided data on *age* (in years), *gender* (male/female), highest attained *level of education* as an indicator of SEP, self-reported number of *medical or psychiatric diagnosis*, and whether participants *attended all seven SCC meetings* (yes/no) and used *pharmacotherapy* during their current quit attempt (yes/no).

SEP is a multifaceted concept in which income, level of education, and occupation play a role [30]. Educational level is often used in smoking research to measure SEP, because it has been found to be a better indicator of risk of smoking than the other compartments of SEP [31, 32]. Highest attained *educational level* had four levels: Primary education (in Dutch: basisschool), lower secondary education (in Dutch: lbo, mavo, vmbo, mbo-1, havo-onderbouw), higher secondary education (in Dutch: havo, vwo, mbo 2–4), and tertiary education (in Dutch: hbo, wo).

#### Smoking characteristics

Sinefuma also provided information on participants’ smoking characteristics: *smoking within 30 min after waking* (yes/no) and *tried to stop before* (yes/no). The number of *pack years* was calculated by the number of daily cigarettes divided by 20 and multiplied by the number of years smoked. This variable ‘pack years’ was categorized into six groups: less than 10, between 11 and 20, between 21 and 30, between 31 and 40, between 41 and 50, and more than 50.

#### Smoking outcomes

Data on whether participants smoked during the intervention period was assessed by a repeating question in every step in the app *(‘Did you smoke?’ (yes/no)’)*. This variable was made dichotomous by ‘No’ (did not smoke at all) and ‘Yes’ (did smoke at least once or more).

Lastly, Sinefuma provided data on abstinence after four weeks and one year after the quit date (yes/no). Ideally, a CO measurement was performed by Sinefuma to measure abstinence four weeks after the quit date. However, this was not possible in the COVID groups due to COVID-related safety measures. Instead, abstinence at four weeks after the quit date was self-reported according to the Russel standard (i.e., no puffs, for the last two weeks). To measure if participants were still abstinent one year after the quit date (yes/no), Sinefuma called all participants and asked if they were still abstinent since the quit date. This included the Russel standard according to which a maximum of five cigarettes may be smoked in the last fifty weeks after the quit attempt for a successful quit attempt. When participants could not be reached, the abstinence was recorded as ‘no’.

#### App adherence

*Number of steps completed* was used as an indicator of app adherence in the analyses, as this variable shows best how much of the intervention was delivered to participants. The *number of steps completed* ranges between 0 and 22. This variable was categorized into (1) fewer than 4 steps, (2) 4 to 12 steps, and (3) more than 12 steps. This division was chosen because there are 3 preparation steps, 9 daily steps and 10 weekly steps. However, not all steps were mandatory, so it is possible that a participant who, for example, last completed step 5, only completed 3 steps in total. This will result in number of steps completed category 1) fewer than 4 steps.

### Ethics

Prior to data collection, all participants gave electronic informed consent for participating in the study. All data was securely stored in the Research Memory of the Department of Cardiology of the Leiden University Medical Center and retained for 15 years as required by law. The research team adhered to the requirements for privacy and confidentiality as stated in the Privacy Statement of the Leiden University Medical Center and the GDPR. The study was cleared for ethics by Medical Ethics Committee Leiden Delft The Hague (N20.137). Participation in the study was voluntary and participants received a gift voucher worth twenty euros as compensation, if they had completed the questionnaires [29]. Trainers were compensated with a €40 gift card for participant recruitment.

### Statistical analysis

*Preliminary analyses*. Before investigating the research questions, we performed attrition analyses. Specifically, chi-square tests were used to assess whether intervention group participants for whom SCC data could be linked to app log data differed on background characteristics compared to those for whom data could not be linked. For the variable age, an independent samples t-test was used.

Percentages and means were used to describe sociodemographic and smoking characteristics, as well as app adherence.

*Research questions*. To test the association between app adherence (steps completed) as the dependent variable and participant characteristics as independent variables, we applied a univariable ordinal logistic regression analysis for each predictor variable separately (RQ1). This model was used because the number of steps is a categorical variable with implied order. With this model an odds ratio (OR) is calculated, reflecting the multiplicative change in the odds of being in a higher category on the dependent variable for every one unit increase on the independent variable. Hereafter, a multivariable ordinal logistic regression including significant (*p* ≤ 0.05) variables from the univariable analysis was performed. Cases with missing variables were excluded listwise from the respective analysis. To test the association between the number of steps completed in the StopCoach app (independent variable) and smoking abstinence after four weeks and one year (dependent variable), a chi-square test was performed (RQ2).

All statistical analyses were performed in SPSS version 26.

## Results

### Attrition results

Table 1 shows the results of the attrition analysis. No significant differences in demographic and smoking characteristics, nor abstinence rates at 4 weeks and 1 year were found between study participants for whom user data was and was not available. Age did not differ between study participants from whom user data was (M = 52.8, SD = 12.3) and was not available (M = 52.1, SD = 12.6; t(222) = -0.42, *p* = 0.77).

**Table 1:** Descriptives and chi-square results from users with missing log data compared to users with log data

Variable	Total (%)		User data						
			Yes (%)		No (%)		X^2^		
							*F*	*df*	*p*
**N**	226	(100)	132	(58.4)	94	(41.6)			
**Demographic characteristics**									
**Gender**							3.0	1	0.08
Male	79	(35.0)	40	(30.3)	39	(41.5)			
Female	147	(65.0)	92	(69.7)	55	(58.5)			
**Educational level**							4.3	3	0.23
Primary/Lower secondary	58	(25.7)	29	(22.0)	29	(30.9)			
Upper secondary	71	(31.4)	40	(30.3)	31	(33.0)			
Tertiary or higher	63	(27.9)	43	(32.6)	20	(21.3)			
Unknown	34	(15.1)	20	(15.2)	14	(14.9)			
**Number of medical or psychiatric diagnose**							2.5	4	0.65
0	75	(33.2)	45	(34.1)	30	(31.9)			
1	37	(16.4)	24	(18.2)	13	(13.8)			
2	34	(15.0)	21	(15.9)	13	(13.8)			
3	43	(19.0)	23	(17.4)	20	(21.3)			
≥4	37	(16.4)	19	(14.4)	18	(19.1)			
**Pharmacotherapy during current quit attempt**							1.1	1	0.29
Yes	153	(67.7)	93	(70.5)	60	(63.8)			
No	73	(32.3)	39	(29.5)	34	(36.2)			
**Attended all Sinefuma SCC meetings**							0.0	1	0.94
Not all meetings	39	(17.3)	23	(17.4)	16	(17.0)			
All meetings	187	(82.7)	109	(82.6)	78	(83.0)			
**Smoking characteristics**									
**Pack years at start SCC**							6.6	5	0.25
≤10	38	(16.8)	28	(21.2)	10	(10.6)			
11–20	31	(13.7)	14	(10.6)	16	(17.0)			
21–30	36	(15.9)	25	(18.9)	11	(11.7)			
31–40	32	(14.2)	18	(13.6)	14	(14.9)			
41–50	22	(9.7)	14	(10.6)	8	(8.5)			
>50	18	(8.0)	12	(9.1)	5	(5.3)			
Unknown	49	(21.7)	21	(15.9)	30	(31.9)			
**Abstinence**									
**4 week abstinence**							0.32	1	0.57
Yes	154	(68.7)	88	(66.7)	66	(70.2)			
No	72	(31.9)	44	(33.3)	28	(29.8)			
**1 year abstinence**							0.05	1	0.82
Yes	75	(33.2)	43	(32.6)	32	(34.0)			
No	151	(66.8)	89	(67.4)	62	(66.0)			

### Preliminary analyses

Table 2 provides descriptive information of intervention group participants for whom app data could be linked to data from the SCC database (*N* = 132), both for the entire group and separately for each app adherence category for steps completed. Woman were in the majority and the mean age was 52.8 years. Higher secondary and tertiary education were the most common educational levels of participants. Information about participants’ smoking behaviour can also be found in Table 2. Most participants had between 21 and 30 pack years, smoked within 30 min after waking up, and did not smoke during the quit attempt as assessed by the StopCoach app in each step.

**Table 2:** Demographic and smoking characteristics, and abstinence: descriptive statistics (*N* = 132)

Variable	Total (%)		App adherence: steps completed					
			< 4 (%)		4–12 (%)		> 12 (%)	
**N**	132	(100)	44	(33.3)	46	(34.8)	42	(31.8)
**Demographic characteristics**								
**Age**								
Mean	52.8		50.5		51.7		56.4	
SD	12.3		13.1		12.7		10.2	
**Gender**								
Male	40	(30.3)	14	(31.8)	17	(37.0)	9	(21.4)
Female	92	(69.7)	30	(68.2)	29	(63.0)	33	(78.6)
**Educational level**								
Primary/Lower secondary	29	(22.0)	8	(18.2)	14	(30.5)	7	(16.7)
Upper secondary	40	(30.3)	15	(34.1)	11	(23.9)	14	(33.3)
Tertiary or higher	43	(32.6)	11	(25.0)	15	(32.6)	17	(40.5)
Unknown	20	(15.2)	10	(22.7)	6	(13.1)	4	(9.5)
**Number of medical or psychiatric diagnose**								
0	45	(34.1)	14	(31.8)	18	(39.1)	13	(31.0)
1	24	(18.2)	7	(15.9)	10	(21.7)	7	(16.7)
2	21	(15.9)	5	(11.4)	7	(15.2)	9	(21.4)
3	23	(17.4)	11	(25.0)	2	(4.3)	10	(23.8)
≥4	19	(14.4)	7	(15.8)	9	(19.6)	3	(7.1)
**Stopped before**								
Yes	114	(86.4)	40	(90.9)	40	(87.0)	34	(81.0)
No	18	(13.6)	4	(9.1)	6	(13.0)	8	(19.0)
**Pharmacotherapy during current quit attempt**								
Yes	93	(70.5)	30	(68.2)	32	(96.6)	31	(73.8)
No	39	(29.5)	14	(31.8)	14	(30.4)	11	(26.2)
**Attended all Sinefuma SCC meetings**								
Not all meetings	23	(17.4)	12	(27.3)	6	(13.0)	5	(11.9)
All meetings	109	(82.6)	32	(72.7)	40	(87.0)	37	(88.1)
**Smoking characteristics**								
**Pack years at start SCC**								
≤10	28	(21.2)	9	(20.5)	11	(23.9)	8	(19.0)
11–20	14	(10.6)	5	(11.4)	5	(10.9)	4	(9.5)
21–30	25	(18.9)	6	(13.6)	12	(26.2)	7	(16.7)
31–40	18	(13.6)	4	(9.1)	7	(15.2)	7	(16.7)
41–50	14	(10.6)	5	(11.4)	4	(8.7)	5	(11.9)
>50	12	(9.1)	3	(6.8)	2	(4.3)	7	(16.7)
Unknown	21	(15.9)	12	(27.2)	5	(10.9)	4	(9.5)
**Smoking early**								
Yes	118	(89.4)	40	(90.9)	38	(82.6)	40	(95.2)
No	14	(10.6)	4	(9.1)	8	(17.4)	2	(4.8)
**Smoked during StopCoach use**								
Yes	25	(18.9)	6	(13.6)	19	(41.3)	12	(28.6)
No	95	(72.0)	38	(86.4)	27	(58.7)	30	(71.4)
**Abstinence**								
**4 week abstinence**								
Yes	88	(66.7)	24	(54.5)	31	(67.4)	33	(78.6)
No	44	(33.3)	20	(45.5)	15	(32.6)	9	(21.4)
**1 year abstinence**								
Yes	43	(32.6)	8	(18.2)	10	(21.7)	25	(59.5)
No	89	(67.4)	36	(81.8)	36	(78.3)	17	(40.5)

### Sociodemographic and smoking characteristics and app adherence

Table 3 presents the results of the univariable and multivariable ordinal logistic regression analysis for the association between sociodemographic and smoking characteristics and app adherence. The univariable models showed that participants who were older and those who had attended all Sinefuma meetings were significantly more likely to complete more steps in the StopCoach app. Other variables were not significantly associated with number of steps completed in the StopCoach app. In the multivariable model with age and SCC attendance, higher age and attending all SCC meetings were still significantly associated with completing more steps in the StopCoach app. To assess the robustness of the findings, we conducted a sensitivity analysis treating SCC attendance as a continuous variable, which yielded consistent results. Specifically, participants who were older (*p* = 0.03) and those who had attended all Sinefuma meetings (*p* = 0.05) were significantly more likely to complete more steps in the StopCoach app.

**Table 3:** Ordinal logistic regression analysis for the association between participant characteristics and the amount of completed steps in the stopcoach app

Variable	ORs with 95% CI					
	Univariable			Multivariable		
	OR	95% CI	*p*-value	OR	95% CI	*p*-value
**Age**	1.03	(1.00-1.06)	**0.03**	1.03	(1.00-1.06)	**0.02**
**Gender**						
Male	Ref					
Female	0.71	(0.36–1.39)	0.31			
**Educational level**						
Primary/Lower secondary	Ref					
Upper secondary	1.01	(0.42–2.42)	0.98			
Tertiary or higher	1.49	(0.64–3.47)	0.36			
**Number of medical or psychiatric diagnosis**						
0	Ref					
1	1.05	(0.43–2.56)	0.91			
2	1.63	(0.63–4.25)	0.32			
3	0.93	(0.35–2.50)	0.88			
≥4	0.68	(0.26–1.76)	0.42			
**Stopped before**						
No	Ref					
Yes	1.89	(0.75–4.77)	0.18			
**Pharmacotherapy**						
No	Ref					
Yes	1.22	(0.61–2.42)	0.57			
**Attended all Sinefuma SCC meetings**						
No	Ref			Ref		
Yes	2.35	(0.99–5.59)	**0.05**	2.63	(1.06–6.54)	**0.04**
**Pack years**						
≤10	Ref					
11–20	0.92	(0.28-3.00)	0.89			
21–30	1.18	(0.45–3.13)	0.73			
31–40	1.60	(0.54–4.74)	0.40			
41–50	1.09	(0.33–3.63)	0.89			
>50	2.71	(0.71–10.38)	0.15			
**Smoking early**						
No	Ref					
Yes	1.32	(0.51–3.44)	0.57			
**Smoked during app use**						
Yes	Ref					
No	1.71	(0.86–3.37)	0.12			

### App adherence and abstinence rates

A chi-square test of independence showed no statistically significant association between the number of completed steps and 4-week abstinence, X² (2)  = 5.60, *p* = 0.06, Cramer’s V = 0.21. However, descriptively, participants who completed more steps appeared more likely to be abstinent (78.6% abstinent when > 12 steps completed) than those who completed fewer steps (67.4% and 54.5% abstinent when 4–12 and < 4 steps completed, respectively). This association became significant at one year follow-up, *X*^*2*^ (2)[ = 20.5, *p* = < 0.001, *Cramer’s V =* 0.39. More specifically, when completing more than 12 steps, significantly more participants were abstinent 1 year after the quit date (*z =* 3.1, *p* < 0.01) and significantly fewer participants than expected were not abstinent (*z* = -2.1, *p* < 0.05).

## Discussion

### Key findings

This study provided new insight into adherence and associated effectiveness of the smoking cessation app StopCoach, when offered in addition to a group-based SCC program. We examined the association between adherence to the StopCoach app and participant characteristics, as well as the association between app adherence and short- and long-term abstinence. Having a higher age and attending all SSC meetings were uniquely and significantly associated with completing more steps in the StopCoach app. We found no statistically significant association between the number of completed steps and 4-week abstinence. However, descriptively, participants who completed more steps appeared more likely to be abstinent. Importantly, this association became significant at one year after the quit date.

The non-significant positive association of more StopCoach app use with a successful quit attempt is consistent with previous studies in which a dose-response relationship between eHealth intervention use and smoking cessation after 12 months was found [19, 20]. It is surprising, however, that this association was non-significant four weeks after the quit date, but significant one year after the quit date. This can be explained by the fact that the 4-week abstinence is measured while the intervention period of both StopCoach and the group-based SCC program is still ongoing, with the effects of the SCC program preventing detection of further effects of StopCoach. Importantly, StopCoach lasted longer than the SCC program and could have served as relapse prevention. More adherent StopCoach users might have been more successful in quitting simply because they had received more support to quit. Importantly, the current study design does not allow for establishing causal relationships. It is possible that people who were more likely to quit successfully (e.g., because of higher motivation to quit) were also more likely to be more adherent to the StopCoach app [29].

Low adherence is a problem in eHealth interventions [23]. Convincing people to use StopCoach more often during and after regular SCC, may have a positive effect on the success of quit attempts, even in the long-term. One way to reach this could be to make the app itself more engaging to use, for example by incorporating serious gaming elements [33]. Better alignment between StopCoach and SCC could also potentially help to increase app adherence. For example, ensuring that the information provided in the app aligns with what was discussed during that week’s SCC session. The need in alignment was also shown in the first paper of this study on the effectiveness of using StopCoach in combination with regular SCC [29]. Both participants and Sinefuma coaches reviewed StopCoach positively, but mentioned that better alignment with SCC meetings was necessary [29]. Good integration of smoking cessation apps into usual care is considered promising, as apps can improve motivation and adherence, and facilitate communication and content provision [34].

App adherence is not often measured in research on eHealth interventions, and little research has been done on the association between user characteristics and app adherence. In line with our findings, studies that did consider user characteristics also found that older users were more likely to use an app [21, 35–38]. Possible explanations for this could be that with increasing age, the risk of medical or psychiatric diagnosed conditions increase, which could be an internal motivation to quit and therefore increase the odds to make use of digital smoking interventions. This study, however, shows no associations between packyears nor number of diagnoses and app use. This finding is in line with findings from interviews with SCC trainers who have used StopCoach in combination with SCC [29]. They found StopCoach more suitable for older SCC participants, partly due to the app’s appearance. It could therefore be that the StopCoach app design, originally developed for and with users with low SEP, is also suitable for older StopCoach users. Furthermore, attending SCC may also have a positive impact on the use of StopCoach as the use of StopCoach is recommended there. This was also shown in the first paper on this study, where interviews with SCC trainers showed that they found StopCoach to be a good addition to regular SCC, partly due to the possibility for app users to retrieve information, and the way it was added to SCC (i.e. decreasing intensiveness over time and StopCoach intervention outlasting regular SCC) [29]. This is in line with findings that a combination of digital interventions with usual smoking cessation support strengthens the constituent parts [39]. This result reflects previous research on StopCoach, which found an association between adherence and having a professional coach [27]. In addition, the association between attending all SCC meetings and completing more steps of the StopCoach app could be explained by a higher intention to quit smoking in general. Note that the StopCoach app can also be used separately from SCC. Although previous research on StopCoach shows more app adherence when SCC is also used, StopCoach is also useful when SCC is not used [27]. Despite the fact that a quit attempt under professional guidance gives better results, many people who smoke deliberately choose to quit smoking on their own [40].

One factor that seems to predict more intensive app use in previous studies is higher educational attainment. However, our study found no association between educational attainment and app use. This could be an indication that StopCoach is similarly suitable to use for people with lower and higher SEP. This result also shows that StopCoach can make a positive contribution to reducing SES inequalities in smoking, confirming results from our earlier study [27] which found no significant SEP differences in the rating of StopCoach and abstinence. In addition, no difference in abstinence was found between the high and low SEP subgroups. This result is in contrast with evaluations of many other smoking cessation interventions, which have been proven less suitable for people with lower SEP [18, 27, 38, 41]. This result also means, as indicated by SCC trainers in our first study, that more suitable eHealth smoking cessation apps may exist for higher SEP participants and younger participants [29].

### Strengths and limitations

Strengths of this study are that it evaluates the use of StopCoach, an eHealth intervention for smoking cessation, in real-world practice. We were able to examine app use and relate this to both participant characteristics and abstinence rates at 4 weeks and 1 year after the quit date, However, there are some limitations of this study that need further clarification.

First, part of the SCC data from intervention group participants could not be linked to their StopCoach app user data, such that the sample for the analyses of app use and adherence was smaller than intended. No differences were found in both demographics and abstinence between participants whose user data could or could not be linked. This suggests that the results are representative for the entire intervention group. Additionally, the study might have been underpowered to detect some associations, which were close to significance but not statistically significant. A post hoc power analysis showed that 219 participants would have been required to detect a significant difference in the number of completed steps for 4-week abstinence at *p* < 0.05 with effect size Cramer’s V = 0.21 and a power of 80%. A second limitation of this study is that data were collected during COVID-19 which possibly influenced smoking cessation in general. It is not clear what influence COVID had on smoking behaviour. A Dutch study found that smoking during COVID-19 combined with high stress caused a change in smoking behaviour (both in increase and decrease and another study showed that lockdown and isolation during COVID-19 would actually lead to an increase in smoking [42, 43]. A third limitation of this study is that there may be variables that were not included in the study but could have influenced the use of the intervention, such as eHealth literacy. This variable, and potentially others, were not included in Sinefuma data, and were not added to the other questionnaire due to length constraints for feasibility reasons.

## Conclusion

The main results of this second paper on the study on the effectiveness of the mHealth application StopCoach are that higher age and attending all SCC meetings were significantly associated with completing more steps in the StopCoach app. Educational level was not significantly associated with app adherence, suggesting that StopCoach is not used differently by users with higher or lower SEP. This contrasts with many other studies where interventions fit less to people with low SEP. Participants completing more steps in the StopCoach app had non-significant higher odds of being abstinent 4 weeks after the quit date. One year after the quit date, this association is significant. These results indicate that eHealth interventions such as StopCoach may increase the effectiveness of traditional SCC programs. Users could benefit from further implementation and alignment in SCC motivating them to complete more steps of the StopCoach app, thereby supporting them to quit successfully.

## Data Availability

The datasets during and/or analyzed during the current study available from the corresponding author on reasonable request.

## Abbreviations

CI: Confidence interval
OR: Odd ratio
SCC: Smoking cessation counseling
SD: Standard deviation
SES: Socioeconomic position
